# A horizontal and perpendicular interlaminar approach for intrathecal nusinersen injection in patients with spinal muscular atrophy and scoliosis: an observational study

**DOI:** 10.1186/s13023-024-03278-8

**Published:** 2024-07-15

**Authors:** Chanyan Huang, Yuanjia Zhang, Daniel A. Diedrich, Jiawen Li, Wei Luo, Xu Zhao, Yuting Guo, Yijun Luo, Tao Zhang, Xuan Wang, Wenqi Huang, Ying Xiao

**Affiliations:** 1 Department of Anesthesiology, The First Affiliated Hospital, Sun Yat-Sen University, Guangzhou, China; 2 Department of Anesthesiology and Perioperative Medicine, Mayo Clinic, Rochester, MN USA; 3 Department of Neurology, Peking Union Medical College Hospital, Beijing, China

**Keywords:** Lumbar puncture, Nusinersen, Scoliosis, Spinal muscular atrophy, Ultrasonography

## Abstract

**Background:**

Lumbar puncture is challenging for patients with scoliosis. Previous ultrasound-assisted techniques for lumbar puncture used the angle of the probe as the needle trajectory; however, reproducing the angle is difficult and increases the number of needle manipulations. In response, we developed a technique that eliminated both the craniocaudal and lateromedial angulation of the needle trajectory to overall improve this technique. We assessed the feasibility and safety of this method in patients with scoliosis and identify factors related to difficult lumbar puncture.

**Methods:**

Patients with spinal muscular atrophy and scoliosis who were referred to the anesthesia department for intrathecal nusinersen administrations were included. With a novel approach that utilized patient position and geometry, lumbar puncture was performed under ultrasound guidance. Success rates, performance times and adverse events were recorded. Clinical-demographic and spinal radiographic data pertaining to difficult procedures were analyzed.

**Results:**

Success was achieved in all 260 (100%) lumbar punctures for 44 patients, with first pass and first attempt success rates of 70% (183/260) and 87% (226/260), respectively. Adverse events were infrequent and benign. Higher BMI, greater skin dural sac depth and smaller interlaminar size might be associated with greater difficulty in lumbar puncture.

**Conclusions:**

The novel ultrasound-assisted horizontal and perpendicular interlaminar needle trajectory approach is an effective and safe method for lumbar puncture in patients with spinal deformities. This method can be reliably performed at the bedside and avoids other more typical and complex imaging such as computed tomography guided procedure.

**Supplementary Information:**

The online version contains supplementary material available at 10.1186/s13023-024-03278-8.

## Introduction

Spinal muscular atrophy (SMA) is a genetic motor neuron disease that results in progressive muscular atrophy and weakness [1]. Nusinersen is an antisense-oligonucleotide approved for the treatment of SMA, which increases the expression of survival motor neuron protein and leads to suppression of apoptosis of neurons and subsequently improves muscle strength. Since the drug is unable to cross the blood–brain barrier, it must be repeatedly administered into the cerebrospinal fluid (CSF) by lumbar puncture (LP) [2, 3]. However, patients with SMA commonly develop neuromyopathic scoliosis with the axial rotation of the spine and ossification of the interspinous ligament, both of which pose challenges in performing LP.

Real-time fluoroscopy and computed tomography (CT) have been used to facilitate LP in scoliotic patients [4–9]. However, these two modalities require specialized equipment, training, and credentialing to operate. Additionally, repeated appointments for injection under fluoroscopy or CT imaging may impact their availability for other imaging. Lastly, repeated radiation exposure has potential to increase the risk of malignancy [10]. To avoid many of these issues, real-time ultrasound-guided LP could be a useful option. However, the traditional method for performing this technique has its own barriers including specialized training and advanced skills in needle-beam alignment, which can limit its effective use and generalization [11–15].

Traditional ultrasound-assisted LP with the identification of the needle entry point and the angle of needle trajectory have been reported demonstrating shortened procedural time, fewer skin punctures and needle redirections [16, 17]. However, matching the correct needle trajectory to the probe angle is difficult and may require multiple needle passes. This is a major limitation of this technique and a single needle pass is desirable to reduce the risk of complications and improve patients’ satisfaction [18, 19].

To address the majority of these issues, we developed a novel ultrasound-assisted interlaminar approach for LP, which allows for a horizontal and perpendicular needle trajectory (needle insertion without lateromedial and craniocaudal angulation). This technique is easy to grasp and is reproducible for the intrathecal nusinersen delivery in SMA patients with difficult spine anatomy.

The current literature is inconsistent in regard to the predictors of difficult LP in scoliotic patients. Carrera-García and colleagues showed that a Cobb angle greater than 50° and a history of spinal surgery could be used to anticipate difficulty in LP [20, 21]. In contrast, Veiga-Canuto and colleagues found that neither the Cobb angle nor previous spinal surgery appeared to predict the level of difficulty; however, the absence of interlaminar space on pre-procedural CT did [14].

In this study, we describe our novel ultrasound-assisted technique for LP in SMA patients with scoliosis with our primary objective to assess the feasibility and safety of this approach. The secondary objective was to determine the factors associated with difficult LP.

## Methods

This observational study (Ref: [2022]413) was approved by the Institutional Research Ethics Committee. From January 2022 to July 2023, all pediatric and adult SMA patients with scoliosis who were referred to the Department of Anesthesiology for intrathecal nusinersen administration were included. Patients were referred there because of the anticipated difficulty in obtaining intrathecal access or previously failed LP. Exclusion criteria included patients with contraindications for LP and Cobb angle less than 11°. Written informed consent was obtained from all subjects and/or their parents.

###  Preprocedural planning and measurements via CT

When available, previous spinal CT scans were reviewed to assess the feasibility of the LP at various levels and to choose the best level with the largest interlaminar space. The L5/S1 space was considered as a last resort due to the decreased volume of the thecal sac. At the preplanned level, the degree of vertebral rotation [22] and the interlaminar space size were measured using the axial CT scan (Supplemental Digital Content 1, Fig. S1). The Cobb angles were measured using both the thoracic and lumbar centered curves for each patient, with the larger angle used to quantify the severity of spinal curvature, classified as mild (11° to 25°), moderate (25° to 50°), and severe (> 50°) [23]. The lumbar lordosis angle was measured employing Cobb’s method [24].

### Positioning the patients with ultrasound guidance

**Fig. 1:**
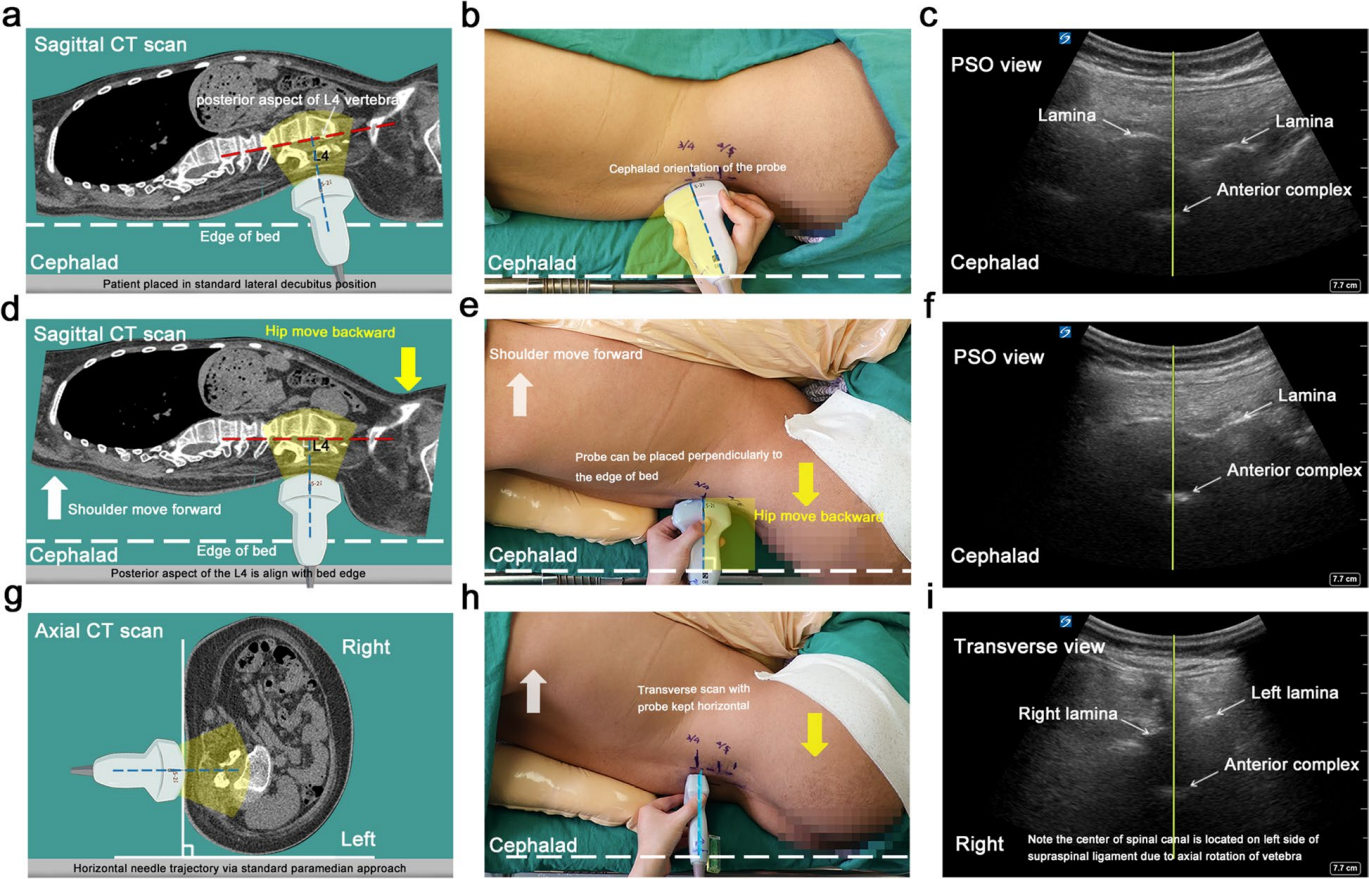
Posture correction guided by the orientation of the ultrasound probe. **a** Sagittal CT view demonstrating the posterior aspect of the L4 vertebra is oblique under standard lateral decubitus position, with the shoulder and the hip being placed at the edge of the bed. **b-c** Note the orientation of the probe relative to the edge of the bed (cephalad direction) to obtain the clear anterior complex image in the paramedian sagittal oblique view. **d** Sagittal CT image demonstrating the posterior aspect of the L4 vertebra being adjusted to align with the edge of the bed following posture correction. **e–f** Modified position with the shoulder moved forward to eliminate the cranial angulation of the probe is required for the clear anterior complex image, ensuring that the needle enters the spinal canal perpendicularly to the edge of the bed. **g** Axial CT view demonstrating the potential horizontal needle trajectory via the standard paramedian approach. **h-i** Note the probe being placed perpendicularly to the edge of the bed and horizontally, with the spinal canal centered. The blue dashed lines indicate the needle trajectory. The white dashed lines indicate the edge of the bed. The white arrow indicates moving the shoulders forward, away from the edge of the bed and the yellow arrow indicates movement of the hips backward, towards the edge of the bed

As most of the patients were unable to sit independently, they were placed in a standard lateral decubitus position with the preplanned puncture site in the dependent position (Fig. 1a, b). The shoulders and hips were kept in line with one another, and the torso as perpendicular to the bed as possible although the coronal plane of the spine varied due to the rotation of the vertebral column. The knees were flexed to overcome the lumbar lordosis that narrowed the interspace between adjacent laminae.

After palpating and marking the position of the spinous processes (SP) of the lumbar spine, a line connecting the SP which we referred to as “SP line” was drawn. Preprocedural ultrasound scanning was then performed using a 5–2-MHz frequency curved probe (Fujifilm Sonosite, Inc., Bothell, WA, USA) by an investigator (YX or CH) who was experienced in neuraxial ultrasound, following a protocol described previously by Dr. Karmakar [25]. While keeping the probe lateral and parallel to the “SP line”, the interlaminar spaces were identified and marked on the skin by counting upwards from the sacrum in the paramedian sagittal oblique (PSO) view. The preplanned intervertebral level was noted and marked and the anterior complex (AC), involving the anterior dura, posterior longitudinal ligament and posterior aspect of vertebral body (Fig. 1b, c) was identified. To obtain optimal visualization of the AC, the direction of the ultrasound beam should be kept as close to perpendicular to the posterior aspect of the vertebral body as possible. The orientation of the posterior aspect of vertebrae segments varied in scoliotic patients, due to the patient’s increased lumbar lordosis or kyphosis.

The patient was positioned on the bed to maintain visualization of the AC and at the same time to ensure the probe was perpendicular to the bed edge. This was done through adjusting the relative position of the shoulders and hips to align the posterior aspect of target vertebra with the edge of bed. The degree of adjustment was dictated by the cephalad or caudad angulation of the probe (Fig. 1a-c). Cephalad angulation of the probe indicated an anterior oblique direction of the posterior aspect of target vertebra. In response the shoulders should were moved forward away from the edge of the bed while the hips were moved backward towards the edge of the bed to eliminate the need of cephalad angulation (Fig. 1d-f). Caudal angulation of the probe indicated a posterior oblique direction of posterior aspect of target vertebra, and, thus, the shoulders were moved backward towards the edge of the bed while the hips moved forward away from the edge of the bed.

### Identification of the needle entry point for a horizontal and perpendicular needle trajectory

**Fig. 2:**
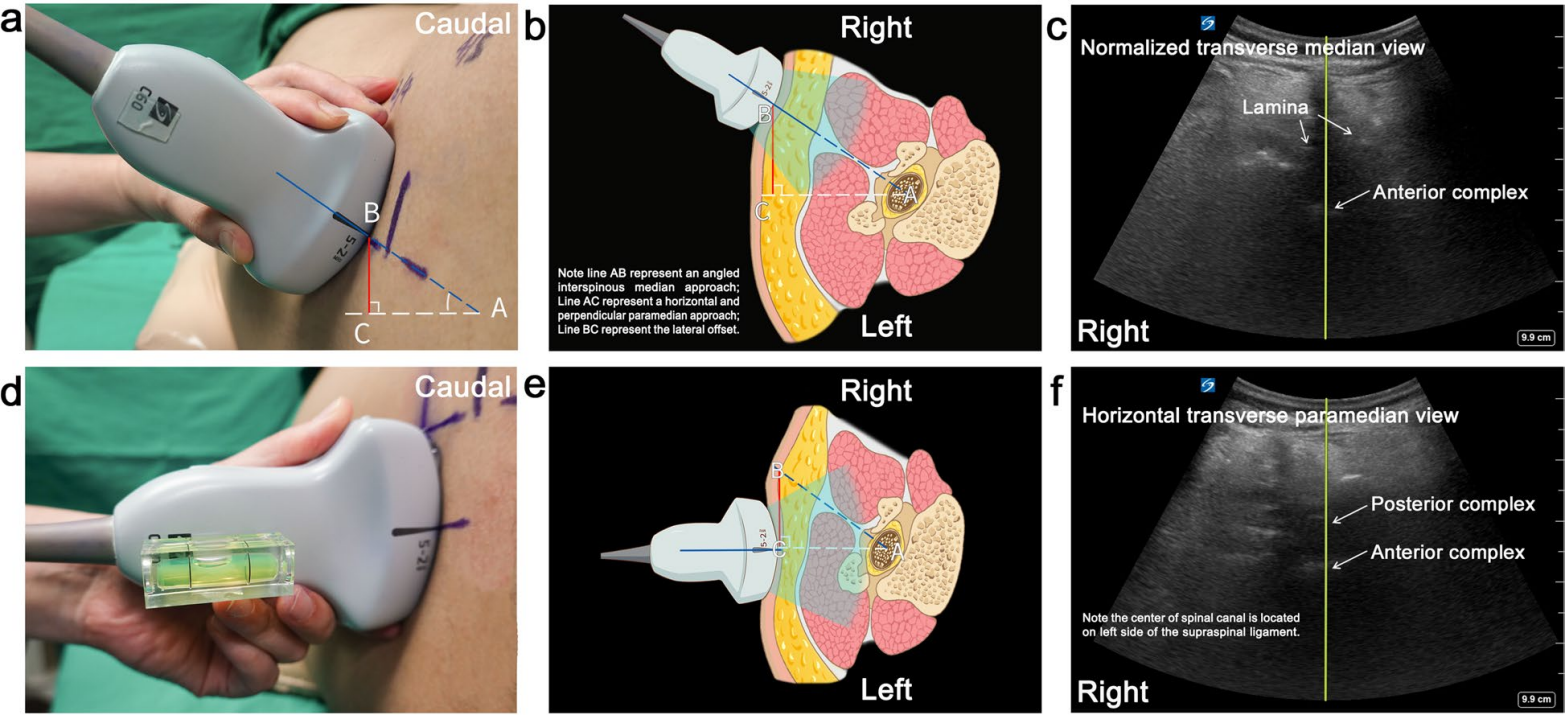
Determination of the needle entry point for a horizontal and perpendicular needle trajectory.** a-c** The probe is rocked to produce the normalized interspinous view with the articular processes or laminae at the same horizontal level, indicating an angled interspinous median approach for lumbar puncture. **d-f** The probe is kept parallel, as verified by attaching a spirit level to obtain the image of the spinal canal with the dural sac centering in the ultrasound screen. Skin marks are made at the midpoint of the probe's long and short edges. The intersection of these two marks is identified as the needle entry point, through which we could direct the needle in the horizontal plane. Point A indicates the center of the spinal canal. Point B indicates the needle entry point for an angled interspinous median approach. Point C indicates the needle entry point for a horizontal needle trajectory via the paramedian interlaminar approach

**Fig. 3:**
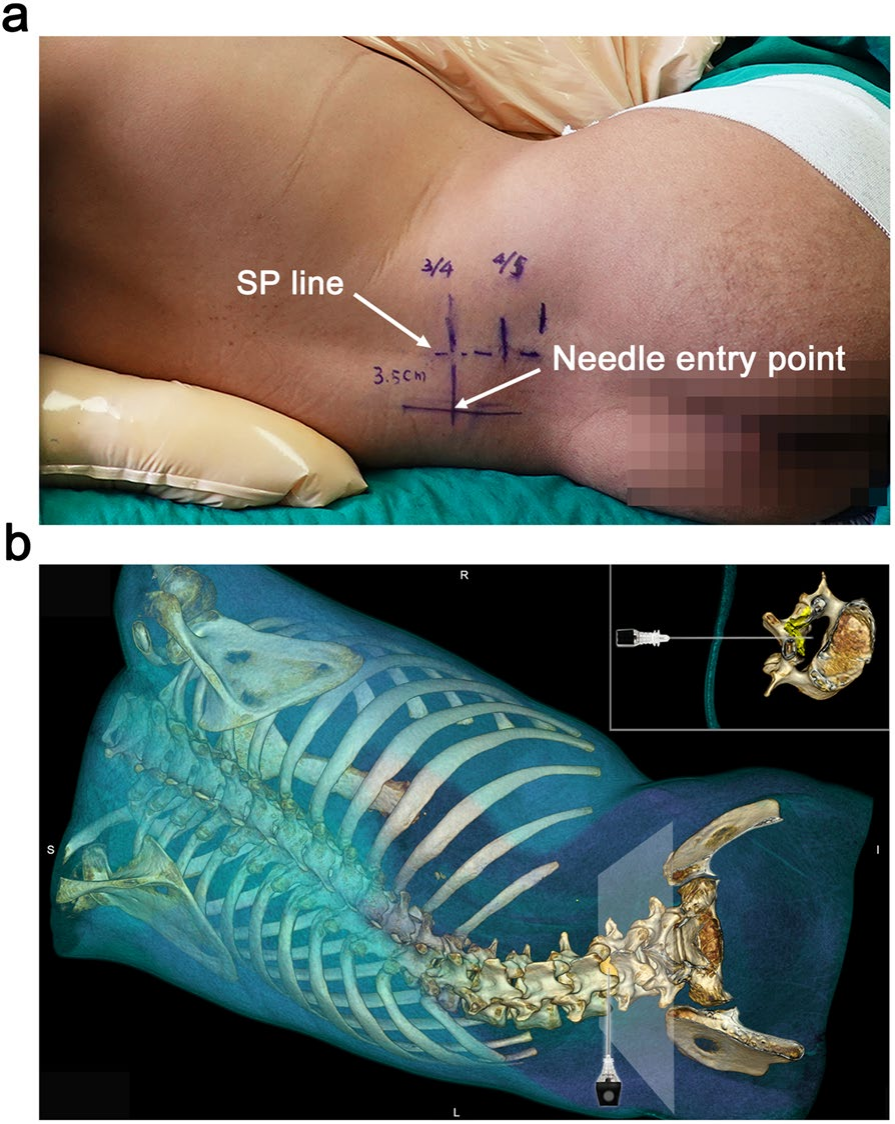
A horizontal needle path demonstrated by CT volume rendering reconstruction images. **a** The determined needle entry point (white arrow) of a SMA patient with scoliosis, 3.5 cm from the spinous process (SP) line. **b** Three-dimensional CT reconstruction in a volume rendering technique of this patient demonstrating the needle directed horizontally to the center of the dural sac on the convex side through the determined needle entry point at the L3 – L4 interspace. SMA, spinal muscular atrophy

The transverse median (TM) scan was then conducted by positioning the probe perpendicular to the long axis of the spine (Fig. 1g-i). If a rocking maneuver of the probe was required to see the laminae or articular processes on both sides at the same level with a clear AC, it indicated a clockwise axial rotation of the target vertebra from caudal-to-cephalic view (Fig. 2a-c). Conventionally, the degree of rocking required to produce the interspinous views was approximately the needle insertion angle via the median approach. To convert this angle onto a horizontal paramedian interlaminar approach, we introduced the concept of a lumbar puncture’s triangle. The right triangle’s hypotenuse was the angled median approach, its base was the horizontal paramedian approach, and its height was the lateral offset from the midline (Fig. 2b & e). To locate the needle insertion point, the probe was held perpendicular to the edge of the bed at the marked interspace and kept horizontal as verified by a spirit level attached on the probe, with the dural sac centered on the ultrasound screen (Fig. 2d-f); note that the thecal sac was at the lateral side of the interspinous ligament. Finally, the needle insertion point was noted and marked by the intersection of the midpoint of the probe’s long edge and short edge – all while keeping the needle horizontal and perpendicular to the bed (Fig. 3).

### Intrathecal injections

After sterile preparation of the skin, 2–5 ml of 2% lidocaine was infiltrated locally and a skin puncture was performed at the marked insertion point. A 25-gauge or 22-gauge needle was inserted horizontally and perpendicular to the edge of the bed to access the subarachnoid space. The needle position was confirmed by successfully aspirating 5 ml of CSF. Nusinersen (5 ml, 12 mg; Spinranza ®; Biogen; Cambridge, MA, USA) was intrathecally injected over 1–3 min.

A video that shows the whole process including the preprocedural ultrasound scan, needle entry site identification, and intrathecal nusinersen administration is available (Supplemental Digital Content 2).

### Outcome measures

Patient demographics and clinical data were obtained from the electronic medical records. A video camera was used to record the process of all LP. Two investigators independently reviewed the video and recorded the following parameters. Discrepancies were resolved by a senior team member.

The primary outcome was the first pass success rate, defined as the needle achieving successful dural puncture through a single skin puncture and no needle redirection [12, 26]. Secondary outcome measures included the following: 1) overall success, with successful puncture defined as a confirmation of the CSF flow through the spinal needle and subsequent nusinersen administration; 2) first-attempt success, defined as the needle achieving successful dural puncture through a single skin puncture; 3) number of needle passes: defined as the number of needle direction adjustments in one puncture, including the first pass; 4) number of needle attempts, defined as the number of any separate skin punctures by a needle; 5) needling time, defined as the time from first needle contact with the skin (beginning with local anesthesia with lidocaine) to visualization of the outflow of CSF; 6) locating time: the time from when the probe was placed on the skin until the skin marking was complete; 7) total procedure time, defined as locating time plus needling time; 8) number of interspace levels tried for successful puncture; 9) adverse events during the procedure and within three post-dural puncture days.

Prior to the study, we devised a classification schema of LP procedural difficulty. A difficult case was defined as a patient who experienced > 2 attempts for at least two LP procedures or > 3 passes for at least two LP procedures.

### Statistical analysis

The normality of data distribution was assessed using the Shapiro–Wilk Test. Continuous data were reported as mean ± standard deviation (SD) with range or median [inter-quartile range (IQR)] with range and analysed with the two-sample t-test or Mann–Whitney U-test, respectively. Categorical variables were reported as number and percentages and analysed with the Pearson Chi-Square test or Fisher’s exact test where appropriate.

All statistical analyses were performed using JMP Pro 16.2.0 software (SAS Institute Inc., Cary, NC, USA). A two-sided *p*-value of less than 0.05 was considered statistically significant.

## Results

During the study period, 49 consecutive SMA patients were referred the Department of Anesthesiology for intrathecal nusinersen administration, of which 5 patients without scoliosis were excluded. The final cohort consisted of 44 patients for analyses, with ages ranging from 4 to 34 years old. Among them, two had previous spine fusion surgery and 40% (18/44) of patients presented with severe scoliosis. The median Cobb’s angle was 39.9° [IQR, 20.8–78.1°; range: 11.0° to 132°]. Only one patient (a four-year old child) regularly received propofol for sedation. Demographic and clinical data of participants are summarized in Table 1.

**Table 1:** Characteristics of patients with scoliosis

Variable	Total (*n* = 44)
Age at first injection, years	15.9 ± 7.3 (range: 4.0 to 34)
Child	11 (25)
Adolescent (10 – 19 years)	20 (45)
Adult	13 (30)
Sex	
Female	23 (52)
Male	21 (48)
BMI, kg.m^−2^	18.8 ± 5.9 (range: 8.7 to 34.7)
SMA type	
2	27 (61)
3	17 (39)
Number of intrathecal nusinersen injections	
1—4	12 (27)
5—8	32 (73)
Non-invasive ventilation support	3 (7)
Sedatives required	1 (2)
Spine abnormalities ^a^	
Previous spinal surgery	2 (5)
Scoliosis	44 (100)
Severity of scoliosis ^b^	
Mild	13 (30)
Moderate	13 (30)
Severe	18 (40)
Cobb’s angle_ max ^c^, °	39.9 [20.8–78.1, range: 11.0 to 132]
Cobb’s angle_ Thoracic centered curve, °	23.9 [13.0–59.1, range: 0 to 132]
Cobb’s angle_ Lumbar centered curve, °	26.9 [14.4–47.9, range: 0 to 124]
Lumbar lordosis angle ^d^, °	43.4 [30.5–58.8, range: 3.1 to 114]
Axial rotation of lumbar vertebrae ^d^, °	
L3	17.6 [7.8–31.1, range: 0 to 84.2]
L4	14.0 [7.4–25.8, range: 0 to 74.1]
L5	13.1 [5.4–25.1, range: 0 to 64.0]

Values are number (proportion), mean ± SD with range, or median [IQR, range]

*Abbreviations*: *BMI* Body mass index, *CT* Computed tomography, *SMA* Spinal muscular atrophy

^a^Spine abnormality may be listed in more than one category; therefore, totals add to more than 100%

^b^Scoliosis is classified based on the maximal Cobb’s angle as mild (11° to 25°), moderate (25° to 50°), or severe (> 50°)

^c^Cobb’s angles were calculated using both the thoracic and lumbar centered curves, with the larger angle being referred to as Cobb’s angle_ max

^d^Variables were measured using computed tomography. Data was missing for one patient who did not undergo spinal CT screening

A total of 260 LP were performed, with an overall success rate of 100%. The first pass and first attempt success rates were 70% (183/260) and 87% (226/260), respectively. Using ultrasound to mark the needle insertion point took a median of 8.5 min [IQR, 7.0–11 min; range, 2.5 to 90 min]. The median needling time required to achieve successful dural puncture was 1.3 min [IQR, 1.0–2.1 min; range, 0.4 to 30 min]. During the procedures, transient paresthesia without post-procedure lumbar radicular pain occurred in 3% (8/260) of LP. The most frequent post-puncture adverse events were lower back discomfort or pain (7%) and headache (4%); none of the patients required any intervention. Details of LP procedures are summarized in Table 2.

**Table 2:** Characteristics and outcomes of lumbar puncture procedures in patients with scoliosis

Variable	Total (*n* = 260)
**Characteristics**	
Intervertebral level of successful puncture	
L2-L3	16 (6)
L3-L4	108 (42)
L4-L5	130 (50)
L5-S1	6 (2)
Needle insertion depth, cm	5.3 [3.7- 6.6, range: 2.0 to 9.0]
Needle entry point deviation from ‘midline’, cm ^a^	1.1 [0.8–1.5, range: 0.4 to 3.5]
**Outcomes**	
First pass success ^b^	183 (70)
Overall success rate	260 (100)
Number of passes	1 [1–2, range: 1 to 18]
Number of attempts^c^	1 [1–1, range: 1 to 6]
First-attempt success	226 (87)
Two attempts required	19 (7)
More than two attempts required	15 (6)
Needling time, min ^d^	1.3 [1.0–2.1, range: 0.4 to 30]
Locating time, min ^e^	8.5 [7.0–11, range: 2.5 to 90]
Total procedure time, min	10 [8.0–12.8, range: 3 to 120]
Number of interspace levels adjustments per procedure	
1	254 (98)
2	6 (2)
Adverse events	
Paresthesia	8 (3)
Lower back discomfort or pain	19 (7)
Headache	10 (4)
Fever	3 (1)
Vomiting/Nausea	2 (1)

Values are number (proportion) or median [IQR, range]

^a^Needle entry point deviation from ‘midline’, defined as the distance from the ‘vertebral midline’ (the line connecting the interspinous ligament and the center of the thecal sac on the ultrasound screen) to the paramedian needle entry point

^b^First pass success, defined as the needle achieving successful dural puncture through a single skin puncture and no needle redirection

^c^Number of attempts, defined as the number of needle insertions through the skin surface

^d^Needling time, defined as the time from first needle contact to the skin (beginning with local anesthesia with lidocaine) to visualization of the outflow of cerebrospinal fluid

^e^Total procedure time, defined as the time from positioning the patient to the outflow of cerebrospinal fluid

Nine patients were categorized as having difficult LP. Compared with the non-difficult group, the difficult group had a higher body mass index (BMI), larger skin-dural sac depth, and narrower interlaminar size (all *P* < 0.05). More patients in the difficult group had an interlaminar space width or height below 4 mm (44% vs. 6%, *P* = 0.012 and 56% vs. 6%, *P* = 0.002, respectively) than those in the non-difficult group (Table 3).

**Table 3:** Characteristics of difficult cases and non-difficult cases with scoliosis

**Variables**	**Non-difficult cases** **(** ***n*** ** = 35)**	**Difficult cases** **(** ***n*** ** = 9)**	***P*** ^a^
Age, years	15.2 ± 7.4	18.4 ± 6.7	0.230
BMI, kg.m^−2^	17.7 ± 5.5	23.3 ± 5.6	**0.020**
Sex_ male	17 (49%)	4 (44%)	0.562
SMA type_3	13 (37%)	4 (44%)	0.785
Cobb’s angle_ thoracic, °	24.5 [12.9–59.7]	23.2 [6.6–70.9]	0.977
Cobb’s angle_ lumbar, °	26.7 [16.0–48.7]	27.0 [11.8–64.7]	0.930
Scoliosis_ severe	14 (40%)	4 (44%)	0.735
Lumbar lordosis angle ^b^, °	41.8 [29.7–55.3]	48.7 [31.2–76.9]	0.124
Rotation of lumbar vertebrae ^b^, °	12.8 [5.3–25.2]	16.3 [6.6–56.3]	0.362
Skin-dural sac distance^c^, cm	5.0 [3.7–6.2]	6.5 [5.4 -8.5]	**0.011**
Interlaminar space width ^d^, mm	7.0 ± 2.0	4.5 ± 1.9	**0.003**
Interlaminar space width < 4 mm	2 (6%)	4 (44%)	**0.012**
Interlaminar space height ^e^, mm	7.8 ± 2.4	5.1 ± 3.2	**0.041**
Interlaminar space height < 4 mm	2 (6%)	5 (56%)	**0.002**

Values are number (proportion), mean ± SD, or median [IQR]

*Abbreviations*: *BMI* Body mass index, *SMA* Spinal muscular atrophy

^a^Two-sample t-test used to compare means, Mann–Whitney U-test used to compare medians, and Pearson Chi-Square test or Fisher’s exact test used to compare proportions

^b^Variables were measured using computed tomography. Data was missing for one patient who did not undergo spinal CT screening

^c^Skin-dural sac distance equals to the needle insertion depth

^d^Interlaminar space width, the maximal distance between the center of the ligament flavum and the most medial aspect on the facet joint at the puncture side

^e^Interlaminar space height, the maximal distance between adjacent laminae on the sagittal view reconstructed parallel to the line of the superior spinous process of the intervertebral space and the center of the thecal sac

## Discussion

Neuromuscular scoliosis in SMA patients with hyperkyphosis and/or hyperlordosis, vertebral rotation and spondylodesis, makes LP difficult, even with the aid of ultrasound. We introduced a horizontal and perpendicular interlaminar approach under ultrasound guidance to facilitate intrathecal injection effectively and safely, with 100% success rate and 70% first pass success rate.

To our knowledge, among recent studies on image-guided intrathecal nusinersen injections for SMA patients with scoliosis and/or history of lumbar spinal surgery, ours is the first observational study collecting a relatively large number of LP procedures to describe the preprocedural ultrasound-assisted approach in patients with SMA (Supplemental Digital Content 3, Table S1). The use of ultrasound for LP has been reported to reduce the number of needle passes required for successful neuraxial anesthetic in patients with abnormal spine anatomy; however, the first pass success rate was much lower than that of our patient cohort (50% vs. 70%), although the average cobb angle (12.3°) in their patient cohort was lower than that of ours (39.9°) [17]. Obtaining the optimal angle for needle trajectory was the main reason for differences in success rate.

The easiest needle angle to replicate and maintain on insertion is one that is perpendicular to the back when it was kept vertical and parallel to the edge of the bed. For SMA patients with scoliosis, the vertebral orientation varies from segment to segment due to the altered curvature of the spine and vertebral rotation, consequently necessitating specific craniocaudal and lateromedial angulation of probe to visualize the spinal canal. The patient’s position was adjusted until the probe was perpendicular to the edge of the bed in reference to the craniocaudal angulation of the probe needed for an optimal sagittal view. Vertebral rotation was used to transfer an angled midline approach to a horizontal paramedian approach (Fig. 2b & e). Following this positioning, all that is required of the proceduralist is keeping the needle during insertion in the center of the canal of the transverse scan horizontal and perpendicular relative to the edge of the bed. This technique translates into increased procedure efficiency as our study demonstrates.

A real-time ultrasound-guided technique has been described as a useful option for SMA patients with abnormal spinal anatomy [13, 14, 27–30]. However, the reported first-attempt success rates were much lower than ours (40% or 55% vs. 87%) [13, 27]. Even using real-time guided techniques with the aid of the SonixGPS® system (an electromagnetic needle tracking system) in patients without spinal abnormalities, the first pass and first-attempt rates were lower than ours (57% vs. 70% and 71% vs. 87%, respectively) [31]. We agree with Chin’s opinion [32] that the real-time ultrasound-guided technique for LP to be technically challenging and requires a higher level of skillset to align the ultrasound beam with the needle path and thus does not offer significant benefits. This is likely because the subarachnoid space is deep, and maintaining visibility of the needle is difficult due to the steep insertion angles during in-plane insertions. Hence, real-time guided procedures are usually reserved for experts rather than an inexperienced practitioner.

The median procedural duration in our study was comparable to that reported for CT-guided LP in SMA patients with scoliosis (10 min vs. 9 min; starting point: ultrasound scan vs. CT scout acquisition), and, similar to CT-guided interventions, adverse events were infrequent and mild [6]. Our results suggest that using our novel ultrasound-assisted approach for LP in SMA patients with complex spine anatomy is as fast as CT guidance. This is relevant as this technique can be done anywhere that has a patient bed and in resource constrained health care systems, availably of a CT scan may not possible. Although CT-guided procedures are considered more precise than other imaging techniques for patients with severe spinal deformities, the potential health risks from the accumulation of radiation exposure cannot be overlooked because SMA patients are generally young and successful management requires treatments with six injections in the first year and three injections per year thereafter [10].

Our study indicated that higher BMI, greater skin-dural sac depth, and smaller interlaminar size might be associated with greater difficult in LP. In contrast with the previous studies [20, 33], we showed that the Cobb angle did not appear to be associated with difficult needle insertion. Consistent with previous reports showing LP to be more difficult in obese patients [34], we observed that patients with higher BMI and greater skin-dural sac depth appeared to be more likely to experience more passes. The explanation may be that the compression force of the ultrasound probe is increased to maximize imaging quality when subcutaneous tissues are thick, loose and mobile, leading to the decreased accuracy of markings. Furthermore, even rather small deviations in any direction of the needle, which might occur unnoticeably, would lead to relatively large displacements of the needle tip at deep layers. Our observations suggested that the most challenging spines are those with an interlaminar space width or height of less than 4 mm, indicating increased precision is desired.

Our study has several limitations. First, it was an observational study conducted in a single medical center with small numbers of patients, further multi-center studies are needed to better define the relative benefits of this and other approaches for patients with complex spinal anatomy and instrumentation. Second, while ultrasound imaging and skin markings were performed by two experienced investigators, the actual needle insertion was performed by practitioners with varied experience. Additional research is also needed to determine the learning curve associated with this technique for operators with no previous experience in ultrasound imaging.

## Conclusions

In conclusion, this novel ultrasound-assisted horizontal and perpendicular interlaminar needle trajectory approach is an efficient and safe method for lumbar puncture in patients with scoliosis secondary to SMA. Given its simplicity, low cost, and safety profile, it could be generalized to other settings for both spinal anesthesia and diagnostic lumbar puncture in challenging patients, such as those with abnormal spinal anatomy, obese, and elderly populations.

### Supplementary Information

Supplementary Material 1: Supplemental Digital Content 1. Fig.S1. Measurements of interlaminar space size via preprocedural CT. Fig. S1 Measurements of interlaminar space size via preprocedural CT. (a) Sagittal CT view (reconstructed parallel to the line connecting the spinous process of L3 and the center of the thecal sac) demonstrating the measurement of the interlaminar height (solid yellow line), defined as the maximal distance between the adjacent laminae. (b) Axial CT view at the L3 – L4 interspace showing the measurement of interlaminar width (solid yellow line), defined as the maximal distance between the center of the ligament flavum and the most medial aspect of the facet joint at the preplanned puncture side.

Supplementary Material 2: Supplemental Digital Content 2. Video S1. The whole process of the novel ultrasound-assisted technique for intrathecal nusinersen injection in SMA patients with scoliosis.

Supplementary Material 3: Supplemental Digital Content 3. Table S1. Comparison of technical success and procedure time associated with image-guided intrathecal nusinersen injections for patients with scoliosis in the past five years.

## Data Availability

All study data will be available from the corresponding author on reasonable request.

## Abbreviations

AC: Anterior complex
BMI: Body mass index
CSF: Cerebrospinal fluid
CT: Computed tomography
IQR: Inter-quartile range
LP: Lumbar puncture
PSO: Paramedian sagittal oblique
SD: Standard deviation
SMA: Spinal muscular atrophy
SP: Spinous processes
TM: Transverse median
