# Supplementary figures and images for: A horizontal and perpendicular interlaminar approach for intrathecal nusinersen injection in patients with spinal muscular atrophy and scoliosis: an observational study
Source: Orphanet J Rare Dis. 2024 Jul 15;19:268. doi: 10.1186/s13023-024-03278-8 (PMC11250962; doi:10.1186/s13023-024-03278-8)

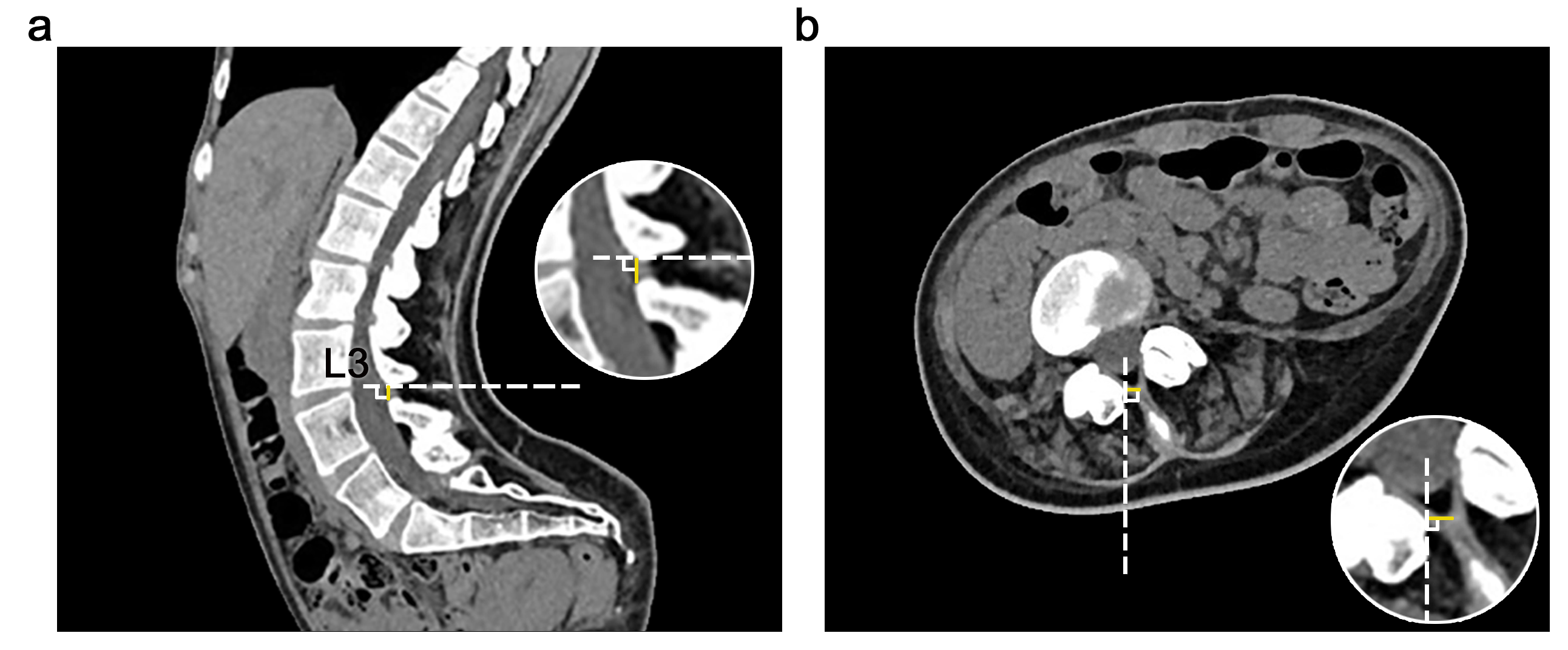

Supplement: Supplementary file 1 — Supplementary Material 1: Supplemental Digital Content 1. Fig.S1. Measurements of interlaminar space size via preprocedural CT. Fig. S1 Measurements of interlaminar space size via preprocedural CT. (a) Sagittal CT view (reconstructed parallel to the line connecting the spinous process of L3 and the center of the thecal sac) demonstrating the measurement of the interlaminar height (solid yellow line), defined as the maximal distance between the adjacent laminae. (b) Axial CT view at the L3 – L4 interspace showing the measurement of interlaminar width (solid yellow line), defined as the maximal distance between the center of the ligament flavum and the most medial aspect of the facet joint at the preplanned puncture side. [file 13023_2024_3278_MOESM1_ESM.tif]
