# Supplementary material for: A horizontal and perpendicular interlaminar approach for intrathecal nusinersen injection in patients with spinal muscular atrophy and scoliosis: an observational study
Source: Orphanet J Rare Dis. 2024 Jul 15;19:268. doi: 10.1186/s13023-024-03278-8 (PMC11250962; doi:10.1186/s13023-024-03278-8)
Supplement: Supplementary file 3 — Supplementary Material 3: Supplemental Digital Content 3. Table S1. Comparison of technical success and procedure time associated with image-guided intrathecal nusinersen injections for patients with scoliosis in the past five years. [file 13023_2024_3278_MOESM3_ESM.pdf]

Table S1. Comparison of technical success and procedure time associated with image-guided intrathecal nusinersen injections for patients with scoliosis in the past five years.

Guidance type, published year and authors	First-attempt success	No. of attempts	Overall success rate	Needling time, min	Total procedure time, min	No. of nusinersen injections	No. of SMA patients	Age, years	Cobb's angle	Other information
**US-assisted; this study**	**87%**	**1 [1-1]**	**100%**	**1.3 [1.0-2.1]**	**10 [8.0-12.8]**	**260**	**44**	**15.9±7.3**	**Median 39.9 (range 11.0-132°)**	**Paramedian interlaminar approach; First-pass success:70%**
US-guided; 2023; Wei et al [1]	55%	1 [1-2]	95%	3 [2–30]	19 [13–56] scanning to completion of injection	20	7	14.1±2.7	Mean ± SD 46.4±33.3	
US-guided; 2022; Snoj et al [2]	-	-	85%	7.8 ± 3.7	-	14	14	32.7±9.1	Mean ± SD 54.6±20.6°	Transforaminal approach
US-guided; 2021; Zanfini et al [3]	-	-	100%	-	11.8 (range, 1.7-28.9)	57	18	44.3 ±11.7	Range 30° - > 50°	In 50% of patients, > 2 attempts needed
US-guided; 2021; Veiga-Canuto et al [4]	-	-	96.8%	-	-	94	18	Median 15.97 (range 6.96–63.5)	Median 53.5 (range 21-87) °	Interlaminar approach

US-guided; 2021; Zhang et al [5]	40%	2 [1-2]	100%	8 [3-15]	-	15	3	25.3±10.3	113±15 °	Paramedian approach
US-guided; 2020; Nagano et al [6]	-	-	100%	-	-	3	1	21	60°	-
Fluoroscopy-guided; 2021; Iwayama et al [7]	82%	-	100%	8.7 [7.5–14.0]	-	38	7	23.9±8.7	76 [20-113] °	Paramedian approach
Fluoroscopy-guided; 2020; Jacobson et al [8]	-	-	100%	Mean fluoroscopy time: 3.8 (range 0.2–15.8)	-	59	12	Mean 21.5 (range, 11–37)	-	Transforaminal; posterior spinal fusion or interlaminar osseous fusion
Combined fluoroscopy- and US-guided; 2022; Berde et al [9]	-	-	100%	-	-	54	6	Range 11-52	-	-
CT-guided; 2022; Salapura et al [10]	94%	-	100%	-	62 ± 25	108	20	Median 33.5 (range, 20–62)	85% > 40°	-
CT-guided; 2021; Rosiak et al [11]	-	-	100%	-	23 [19-30] vs. 28 [24-3]	65	18	28 [22-31] vs. 32 [24-34]	-	Low- vs. standard-dose

CT-guided; 2020; Cordts et al [12]	96%	Mean 1.04	100%	-	Median 9; mean 10.6	53	11	Mean 33 (range 16–46)	-	At least one adverse event: 20.8%
CT-guided; 2020; Spiliopoulos et al [13]	95%	-	-	-	-	20	5	31±9	> 50°	Transforaminal
CT-guided; 2021; Weaver et al [14]	97% succeed at the initial spinal level	-	100%	-	-	200	28	24.1±9.8 (range, 10–51)	Osseous fusion or spinal instrumentation	Transforaminal; 93.5% CT-guided; 6.5% fluoroscopic-guided.
CT-guided; 2019; Wurster et al [15]	-	1.16±0.7	100%	-	41.98±19.88	57	12	28.8±16.2	-	-
CT-guided; 2019; Bortolani et al [16]	-	-	98%	-	-	47	12	30±12.5	> 50°	Interlaminar vs. transforaminal approach
CT-guided; 2018; Weaver et al [17]	-	-	-	Mean fluoroscopy time: 1.9 (range, 0.6–7.1)	-	15	4	Mean 15 (range 13–17)	-	Spinal fusion

Reference:

1. Wei C, Liang Z, Wu Y, et al. Ultrasound-guided interlaminar approach for nusinersen administration in patients with spinal muscular atrophy with spinal fusion or severe scoliosis. *Orphanet J Rare Dis.* 2023;18(1):30.
2. Snoj Z, Salapura V. Ultrasound-guided transforaminal approach for nusinersen delivery in adult spinal muscle atrophy patients with challenging access. *Muscle Nerve.* 2022;65(5):585-589.
3. Zanfini BA, Catarci S, Patanella AK, et al. Ultrasound assisted lumbar intrathecal administration of nusinersen in adult patients with spinal muscular atrophy: A case series. *Muscle Nerve.* 2021;64(5):594-599.
4. Veiga-Canuto D, Cifrian-Perez M, Pitarch-Castellano I, Vazquez-Costa JF, Aparici F. Ultrasound-guided lumbar puncture for nusinersen administration in spinal muscular atrophy patients. *Eur J Neurol.* 2021;28(2):676-680.
5. Zhang J, Cui X, Chen S, Dai Y, Huang Y, Zhang S. Ultrasound-guided nusinersen administration for spinal muscular atrophy patients with severe scoliosis: an observational study. *Orphanet J Rare Dis.* 2021;16(1):274.
6. Nagano T, Sakura S, Imamachi N, Saito Y. Ultrasound-assisted intrathecal injection of nusinersen in a patient with severe vertebral deformity: a case report. *JA Clin Rep.* 2020;6(1):61.
7. Iwayama H, Wakao N, Kurahashi H, et al. Administration of nusinersen via paramedian approach for spinal muscular atrophy. *Brain Dev.* 2021;43(1):121-126.
8. Jacobson JP, Cristiano BC, Hoss DR. Simple Fluoroscopy-Guided Transforaminal Lumbar Puncture: Safety and Effectiveness of a Coaxial Curved-Needle Technique in Patients with Spinal Muscular Atrophy and Complex Spines. *AJNR Am J Neuroradiol.* 2020;41(1):183-188.

9. Berde C, Formanek A, Khan A, et al. Transforaminal lumbar puncture for spinal anesthesia or novel drug administration: a technique combining C-arm fluoroscopy and ultrasound. *Reg Anesth Pain Med.* 2022.

10. Salapura V, Snoj Z, Leonardis L, Koritnik B, Kostadinova V. Cone-beam computed tomography guided nusinersen administrations in adult spinal muscular atrophy patients with challenging access: a single- center experience. *Radiol Oncol.* 2022;56(3):319-325.

11. Rosiak G, Lusakowska A, Milczarek K, et al. Ultra-low radiation dose protocol for CT-guided intrathecal nusinersen injections for patients with spinal muscular atrophy and severe scoliosis. *Neuroradiology.* 2021;63(4):539-545.

12. Cordts I, Lingor P, Friedrich B, et al. Intrathecal nusinersen administration in adult spinal muscular atrophy patients with complex spinal anatomy. *Ther Adv Neurol Disord.* 2020;13:1756286419887616.

13. Spiliopoulos S, Reppas L, Zompola C, et al. Computed-tomography-guided transforaminal intrathecal nusinersen injection in adults with spinal muscular atrophy type 2 and severe spinal deformity. Feasibility, safety and radiation exposure considerations. *Eur J Neurol.* 2020;27(7):1343-1349.

14. Weaver JJ, Hallam DK, Chick JFB, et al. Transforaminal intrathecal delivery of nusinersen for older children and adults with spinal muscular atrophy and complex spinal anatomy: an analysis of 200 consecutive injections. *J Neurointerv Surg.* 2021;13(1):75-78.

15. Wurster CD, Winter B, Wollinsky K, et al. Intrathecal administration of nusinersen in adolescent and adult SMA type 2 and 3 patients. *J Neurol.* 2019;266(1):183-194.

16. Bortolani S, Stura G, Ventilii G, et al. Intrathecal administration of nusinersen in adult and adolescent patients with spinal muscular atrophy and scoliosis: Transforaminal versus conventional approach. *Neuromuscul Disord.* 2019;29(10):742-746.

17. Weaver JJ, Natarajan N, Shaw DWW, et al. Transforaminal intrathecal delivery of nusinersen using cone-beam computed tomography for children with spinal muscular atrophy and extensive surgical instrumentation: early results of technical success and safety. *Pediatr Radiol.* 2018;48(3):392-397.
